# Porcine Circovirus Type 2 (PCV2) Vaccines in the Context of Current Molecular Epidemiology

**DOI:** 10.3390/v9050099

**Published:** 2017-05-06

**Authors:** Anbu K. Karuppannan, Tanja Opriessnig

**Affiliations:** 1 Department of Veterinary Diagnostic and Production Animal Medicine, College of Veterinary Medicine, Iowa State University, Ames, IA 50011, USA; akkarup@iastate.edu; 2 The Roslin Institute and the Royal (Dick) School of Veterinary Studies, University of Edinburgh, Midlothian EH25 9RG, UK

**Keywords:** PCV2, epidemiology, pigs, vaccination

## Abstract

Porcine circovirus type 2 (PCV2) is an economically important swine pathogen and, although small, it has the highest evolution rate among DNA viruses. Since the discovery of PCV2 in the late 1990s, this minimalistic virus with a 1.7 kb single-stranded DNA genome and two indispensable genes has become one of the most important porcine pathogens, and presently is subjected to the highest volume of prophylactic intervention in the form of vaccines in global swine production. PCV2 can currently be divided into five different genotypes, PCV2a through PCV2e. It is well documented that PCV2 continues to evolve, which is reflected by changes in the prevalence of genotypes. During 2006, commercial vaccines for PCV2 were introduced on a large scale in a pig population mainly infected with PCV2b. Since 2012, the PCV2d genotype has essentially replaced the previously predominant PCV2b genotype in North America and similar trends are also documented in other geographic regions such as China and South Korea. This is the second major PCV2 genotype shift since the discovery of the virus. The potential increase in virulence of the emergent PCV2 genotype and the efficacy of the current vaccines derived from PCV2a genotype against the PCV2d genotype viruses has received considerable attention. This review attempts to synthesize the understanding of PCV2 biology, experimental studies on the antigenic variability, and molecular epidemiological analysis of the evolution of PCV2 genotypes.

## 1. Introduction

Infectious disease plays an important role in pig production and prevention is often essential to minimize economic losses. Since the discovery of porcine circovirus type 2 (PCV2) in 1998 [1,2], this small, circular, non-enveloped DNA virus is recognized as one of the most important pathogens of the pig population worldwide. Porcine circovirus (PCV) was first observed as a contaminant in pig kidney cell line in 1974, and in 1982 the 17-nm single-stranded DNA virus with a circular genome was described in more detail [3,4]. The initial name of the virus, PCV, was changed to PCV type 1 (PCV1) in 1998 [5] to differentiate this non-pathogenic virus type from its pathogenic variant PCV2. For many years, PCV1 was considered widespread as antibodies to this virus were found in farmed pigs as well as wild boars, however, no disease association was noted [6,7]. A number of field surveys and experimental inoculations of PCV1 were reported from Canada, the UK and continental Europe, which all showed the absence of pathogenesis in pigs infected with PCV1. In the mid-1990s, the novel PCV2 with a restriction fragment length pattern (RFLP) of 422 was identified and subsequently associated with post-weaning multi-systemic wasting syndrome (PMWS) in Canada [1]. PMWS is characterized by poor weight gain, wasting and general symptoms such as dyspnea, pallor, diarrhea and icterus [8]. These initial finding led to the almost simultaneous identification of PCV2 in diseased pigs in different geographical regions including North America, the UK and France [2]. All these viruses had more than 95% genetic similarity, but were different from PCV1 and hence were named PCV2 [5]. Subsequent studies on the prevalence of PCV2 in the wild pig population indicated its ubiquitous nature [9,10]. A picture of PCV2 as a pig pathogen commonly present in association with other viruses or bacteria became obvious [11,12]. Apart from PMWS, many PCV2 infection-associated clinical conditions such as respiratory symptoms, congenital tremors, enteritis, dermatitis, nephropathy and reproductive issues were described and later grouped as porcine circovirus-associated diseases (PCV-AD) in North America [13] and porcine circovirus diseases (PCVD) in Europe [14]. High PCV2 viremia and viral load in tissues, granulomatous inflammation, depletion of lymphocytes and dysfunction of the lymphoid system causing immunosuppression were characterized as the hallmarks of severe PCV2 infection [15,16,17,18]. Defying Koch’s postulates, experimental reproduction of PCV-AD proved to be difficult and inconsistent and PCV2 was acknowledged, amidst skepticism, as necessary but not sufficient to elicit PCV-AD [12,19,20]. The importance of co-infection or at least a mitogenic trigger to host lymphocytes was understood to be an essential part of the development of severe PCV-AD [21,22]. Experimental co-infection of pigs with PCV2 along with other common swine pathogens consistently resulted in PMWS [12,23,24,25,26]. The first commercial vaccines became available in 2004 in Europe and in 2006 in North America, and have since received wide acceptance among pig farmers worldwide. The decrease in morbidity and improved production efficiency after the adoption of PCV2 vaccines unambiguously emphasized the adverse impact of PCV2 on the health of pigs [27]. PCV2 vaccines are now the single most-selling prophylactic agent in porcine husbandry. Besides clinical disease, the impact of sub-clinical infection of PCV2 on the health of farmed pigs and production parameters has been documented [28]. A wealth of knowledge of various aspects of PCV2 such as its evolution and phylogeny, immune response, interaction of viral proteins with host cellular proteins, and efficacy of its vaccines has been accumulated. This review will focus on the recent developments in antigenic variability, molecular epidemiology and diagnosis of PCV2 infections as well as current challenges in controlling PCV2 infections.

## 2. Virus Replication and Genes

The genome architecture of PCV1 and PCV2 is very minimalistic; among the seven predicted open reading frames (ORFs), only ORF1 and ORF2, which encode for the replicase (Rep) proteins and the capsid (Cap) protein, respectively, are indispensable for virus propagation [29,30]. The ORF1 gene, essential for the replication of the circoviral genome, is present on the sense strand of the encapsulated single-stranded DNA (ssDNA) genome and produces several splice variants of which Rep and Rep’ are the largest [31,32,33,34]. The genome of PCV2 has a conserved stem loop structure present in diverse ssDNA viruses that infect eukaryotes [35]. The Rep and Rep’ proteins bind to a octanucleotide motif on the genome, near the stem loop structure present proximal to the ORF1 gene, and mediates the genome replication through their nicking and joining enzymatic activity [33,36,37]. However, the Rep proteins do not have any polymerase activity and recruit host DNA polymerases and factors, expressed by host cells during the S-phase of cell cycle, to mediate PCV genome replication by rolling circle replication mechanism [38,39]. The ORF2 gene of PCV2 is transcribed from the complimentary strand in the replicative form of the virus to produce a 233 amino acid capsid protein [32,40,41]. PCV1 and PCV2 are icosahedral virions without envelope, and 60 capsomeres form the virus. Porcine circoviruses are thought to have originated from a recombination event between a plant nanovirus and an animal picornavirus, deduced by the conserved stem loop structure at the origin of replication of circovirus and the homology of Rep proteins [42,43].

Despite its minimalistic design, the search for a specific genetic determinant of pathogenicity or virulence of PCV2 has been elusive. Chimeric viruses with ORF1 from the nonpathogenic PCV1 and ORF2 from the pathogenic PCV2 are not pathogenic and elicit protective immune response against a subsequent challenge with PCV2 [44,45,46,47]. The ORF3 gene, encoded on the antisense strand of the PCV2 genome, potentially encodes a 105-amino acid protein implicated in inducing apoptosis of infected cells and has been ascribed to have a role in PCV2 pathogenesis amidst opposing views [48,49,50,51]. Recently, an interesting study has depicted latent and productive PCV2 infection in the corticomedullary junction of the thymus, leading to the dysregulation of T cell maturation [52]. The study suggests that early life PCV2 infection of the thymus could lead to the recognition of PCV2 as self, later leading to a negative selection of PCV2-specific T cells. In addition, the study also suggests the development of adaptive tolerance in peripheral T cells to PCV2 antigens. Overall, the role of the capsid protein in enhancing the fitness of PCV2 replication at the cellular level and the spread of the virus in the host population, with widespread preexisting seroconversion against PCV2, is arguably an important factor in determining the pathogenicity and virulence of the virus. Experimental evidence shows that subtle changes in the PCV2 capsid protein can increase its fitness at the cellular level and increase its virulence in infected pigs [53,54]. Recent field observations have brought the role of the capsid protein in the molecular epidemiology of PCV2 into light [55,56,57,58,59].

## 3. Molecular Epidemiology

Analysis of PCV1 and PCV2 evolutionary trends estimates the time to most recent common ancestor (TMRCA) approximately to the later part of the nineteenth century or the beginning of the twentieth century, as well as subsequent independent evolution in spite of co-circulation [55,56,57,58,59,60]. A convention on the sub-classification of PCV2 into genotypes was accepted in 2008 based on the then prevalent diversity in the ORF2 nucleotide and a p-distance of 0.035, which was the criteria used to delineate the PCV2 genotypes [61]. To date, studies of PCV2 genomes based on the above criteria identify three major genotypes; PCV2a, PCV2b and PCV2d, and two genotypes; PCV2c and PCV2e, with low prevalence [55,58,62,63,64,65]. Examination of archived tissue revealed that PCV2 was circulating among domesticated pigs as early as the 1960s in the US and Germany, the 1970s in Northern Ireland and Switzerland, and from 1980s in the UK and Denmark [53,66,67,68,69]. Since its discovery, two major changes in the prevalence of circulating genotypes of PCV2 have been observed, which are described as “genotype shifts” [55]. The prevalent PCV2a genotype was replaced by the PCV2b genotype in the mid-2000s, with a purported increase in virulence [55,57,70,71,72]. In recent years, the PCV2d genotype (earlier known as mutant PCV2b) has been increasing in prevalence in major pork producing areas, including the United States, Europe, China, Korea and South America [55,56,58,73]. It is interesting that the first genotype shift slightly predates the widespread vaccination against PCV2, while the recent genotype shift is noticed in the presence of widespread vaccination. The PCV2 is recognized for its estimated evolutionary rate of 1.2 × 10^−3^ substitutions/site/year, the highest among comparable DNA viruses [58,60,74]. It should be noted that ORF2 has a higher rate of evolution than the whole genome of PCV2 [58,65], which could be due to the constraints imposed by deleterious mutations in the ORF1 gene with its alternately spliced variants. Interestingly, the evolutionary rate for PCV1, which displays very low genetic diversity, is estimated to be around 1.15 × 10^−5^ substitutions/site/year [75]. Studies to estimate the putative temporal origin of the major PCV2 genotypes are summarized in Table 1.

Global trade in breeding pigs, semen and pork products have contributed to the worldwide dissemination of PCV2 [55,60] to the extent that PCV2c, for long considered to be confined to Denmark, and PCV2d, thought to be a newly divergent genotype, have been isolated in wild boars in the Brazilian Pantanal [76] and are speculated to have be transmitted to the wild boars by peccaries, which are also documented to harbor the PCV2 virus [77].

The patterns in PCV2 genome evolution could have been driven by factors such as natural mutational bias, restraints on genetic variability owing to the topology of its transcripts and genes in overlapping arrangements [34,40,41], evolution for enhanced replication and transmission fitness at the cellular level [53,54], and evolution under immune selective pressure [59,74], either natural or vaccine-induced [78]. As a related point of much practical implications, some reports indicate that the virulence of the different genotypes is similar, while others contend this view [79,80,81,82]. Isolation of recombinant PCV2 genomes, which must have arisen from co-infecting PCV2 genotypes, is a common event in domestic pigs and wild boars [55,83,84,85,86]. Interestingly, recombination breakpoints are found more frequently in the intergenic regions and to a lesser extent in the ORF1 and ORF2 genes. The ORF2 encoded capsid protein is the primary target for the immune system, hence it is naturally under selective pressure from the immune system, as observed by the higher rate of mutation in this gene [58,61]. The case for PCV2 evolution under vaccine pressure, discussed in the next section, is made by comparing the ORF2 amino acid content of PCV2 isolates from pig populations prior to and after the introduction of vaccines, and also by comparing the isolates from unvaccinated farmed pigs, wild boars, and free ranging pigs [74,87,88,89]. The latter comparison is potentially confounded by limited dataset owing to the scarcity of farms not vaccinating against PCV2 and the free transmission of PCV2 from vaccinated pigs and unvaccinated pigs [74].

## 4. PCV2 Vaccines

The main commercial vaccines available to date are derived from the PCV2a genotype or its capsid protein [13], and are acknowledged as highly successful in decreasing the disease burden found prior to the introduction of the vaccine. The current vaccines are efficient in inducing humoral and cell-mediated immunity against PCV2 [90,91,92]. In North America, the PCV2a genotype has gradually been replaced by the PCV2b genotype since 2005 [72], followed by the recent replacement of the PCV2b genotype by the PCV2d genotype as the most prevalent [59]. Despite the health benefits and production parameter improvements associated with the current PCV2 vaccines and its almost universal use in the United States, PCV2 infection is yet widespread among the vaccinated population. An epidemiological study of serum and tissue samples in 2012 showed 7.7% positive for PCV2a and 8.4% positive for PCV2b viral DNA [89], and approximately one in four pigs showed detectable PCV2 viremia, with a break up of 11.3% PCV2a, 29% PCV2b and 71.8% PCV2d, in a recently published study [59]. Another recent study reports a decrease in the prevalence and viremia level of PCV2a and PCV2b in field pig serum collected from 12 states in the United States in 2012 compared to data from the pre-vaccination period of 2006 [93], indicating that vaccines are effective in controlling the virus load in individual pigs and the PCV2 prevalence on a per site basis. The distribution of PCV2 genotypes continues to evolve in the vaccinated population, as reflected by the change in genotype prevalence to PCV2d [59]. Concurrent infection with PCV2a and PCV2b genotypes is thought to be a potentiating factor in the development of clinical disease and PCV-AD cases, with concurrent PCV2a and PCV2b infections also being observed in the field [94].

The immuno-dominant epitopes on the 233 amino acid capsid protein of PCV2, recognized by antibodies from PCV2-infected pigs, are characterized into distinct A, B, C and D regions, which approximately correspond to amino acid segments 65–87, 113–139, 169–183 and few C-terminal amino acids, respectively [95,96]. Signature motifs on the capsid protein can distinguish between PCV2 genotypes, with the emerging PCV2d genotype harboring an extra lysine residue at the C-terminal end at position 234, and PCV2e coding for 238 amino acids with an additional five amino acids at the C-terminal end [59,97]. The characterization of the atomic structure of the PCV2 virus enabled a better understanding of the previously deduced antigenic domains of the viral capsid protein [98]. The immuno-dominant region spanning amino acids 163–180 in the capsid protein of PCV2 is implicated to function as a decoy epitope, resulting in the production of non-neutralizing antibodies against PCV2 [82,99]. Non-neutralizing antibodies against this epitope region were not elicited by PCV2 vaccines but found in serum from PCV-AD-diagnosed pigs, and this epitope has been suggested as a basis to develop serological assays to predict the protective capacity of antibody response against PCV2. Another study demonstrated the existence of antigenic differences in the virus particle structure between PCV2 genotypes, utilizing panels of neutralizing mouse monoclonal antibodies against epitopes in the capsid [100]. The variation of a single amino acid between PCV2a and PCV2b was able to abolish the neutralizing activity of a monoclonal antibody against the PCV2b genotype [101], and a similar effect was observed in another study [102]. These findings highlight the potential of minor variations in the capsid protein that can cumulatively lead to escape from a vaccine-induced immune response. In line with this, PCV2d, which varies from PCV2a by 23 amino acids, most of which fall in or near the epitope A and the decoy epitope regions, was isolated in many apparent vaccine failure cases worldwide [103,104,105,106,107]. Variations in PCV2d capsid protein were found in the conformational epitope regions, loop BC (epitope A) and loop CT, by in silico analysis, and are thought to cause changes in the antigenic structure on the viral surface, enabling improved binding of the PCV2d virus to the host cell and enabling it to escape preexisting immunity [108]. Earlier experimental evidence also showed that the C-terminal epitope D, which is located on the viral surface, is a target for neutralizing antibodies and, interestingly, the PCV2d genotype has an extra lysine residue at its very C-terminal end [95]. Assuming proper vaccination practices are followed in every instance, the current predominant prevalence of PCV2d, which is estimated to have diverged before the introduction of widespread PCV2 vaccines, could potentially reflect the lack of a thorough immunological protection against this genotype by current vaccines. Molecular epidemiological studies to analyze the evolution of PCV2 strains under vaccine pressure have surmised that the capsid protein amino acids in field isolates display divergence from the antigenic determinants in the PCV2a-based vaccine strains, clearly in the case of PCV2a and less so in the case of PCV2b and PCV2d [74,87,88]. These studies also show that the PCV2 population is shrinking in genetic diversity after the introduction of vaccines, mirroring their decrease in prevalence. However, controlled experimental studies and field trials show that PCV2a-based vaccines and a PCV2b-based vaccine confer adequate cross protection against clinical disease upon challenge with PCV2a, PCV2b and PCV2d genotype viruses and improve average daily weight gain [104,109,110,111,112,113,114,115]. These observations show that the current vaccines are adequate in preventing clinical disease in most instances; however, they may represent a “leaky vaccine” situation. This is a terminology used in the field of vaccinology to refer to vaccines which tend to decrease the transmission rate and infection rate on a per exposure basis, such as in a vaccine trial or in the presence of good biosecurity, but may not confer protection under conditions of repeated exposure and the influence of other cofactors [116]. Therefore, current vaccines may be able to minimize PCV2d replication but not abolish it. Experimental evidence from controlled studies also suggest that a homologous vaccine for PCV2b may be better than a heterologous vaccine in decreasing the viremia after concurrent PCV2a and PCV2b challenge, even if both vaccines prevent the development of lesions [78]. Similar differential viremia or viral shedding upon heterologous genotype challenge following PCV2a- or PCV2b-based vaccination has also been recorded in other studies, which are summarized in Table 2 [109,117].

The current situation warrants more long-term experimentation with homologous vs heterologous PCV2 vaccines and even multi-genotype vaccines with an aim to simultaneously control the emergence of the PCV2d genotype and the reemergence of PCV2a and PCV2b genotypes.

Notable among recent efforts in improving the current vaccines against PCV2 is the development of marker vaccines, which will allow the differentiation of infected and vaccinated pigs, with engineered foreign epitopes at the C-terminal end of the capsid protein and vaccines incorporating the PCV2b capsid protein [70,109,119]. A recent novel vaccine candidate, probably the most innovative PCV2 vaccine candidate yet, was developed by the molecular breeding of ORF2 genes of many PCV2 genotypes and the creation of a mosaic ORF2 gene with 234 amino acids which was cloned into the backbone of PCV1 ORF1 [120]. The selected chimeric virus candidate in this study, PCV1-3cl14, displayed wide heterologous immune response and conferred good protection upon heterologous challenge [120]. However, this study lacks information on how the novel chimeric vaccine candidate compares with other commercial and experimental PCV2a or PCV2b vaccines in eliciting neutralizing antibody response and the prevention of viremia and lesions. In addition, further experiments in this direction, perhaps with a directed engineering of a mosaic virus instead of the traditional random molecular breeding approach, used in the above study, would be very interesting. An improvement of current models of vaccine evaluation strategies, similar to the views of Ragonnet et al. [121], should also be seriously considered in light of factors such as the enzootic nature of PCV2, concurrent multi genotype infections, other immuno-suppressing co-infections such as porcine reproductive and respiratory syndrome virus (PRRSV), classical swine fever virus (CSFV) and the presence of feral and wild reservoirs. The reservoir population is a niche where PCV2 diversification could be still ongoing without vaccine-induced immune pressure. Many other factors which may influence the efficacy of a vaccine such as host genetics, inherent difference among the commercial vaccines, time and frequency of vaccine administration, and interference from maternal antibodies are not commented upon here [119,122].

## 5. Diagnosis

Diagnosis of PCV2 as the principal etiology of disease has been centered on the detection of hallmark histopathological lesions of histiocytic infiltration, lymphoid depletion and associated PCV2 antigens/PCV2 genome [13]. Analysis of histological sections for the amount and distribution of PCV2 in suspected tissues by immunohistochemistry and in situ hybridization is considered the gold standard for a diagnosis of PCV-AD. Currently, many commercial enzyme-linked immunosorbent assay (ELISA) kits to detect PCV2 antigens and PCV2-specific antibodies are available and routinely utilized in diagnostic labs. The ELISAs to detect antibodies against PCV2 are of two types, indirect ELISAs and competitive blocking ELISAs [13]. Prior to the advent of these kits, immunofluorescence assay and immune peroxidase monolayer assay were widely used to detect PCV2-specific antibodies and to identify infective PCV2 viruses [13]. Serological assays to detect PCV2-specific antibodies have been adapted to various platforms including microbead-based assays such as Luminex, which allow for the detection of antibodies against other pathogens simultaneously [123]. As PCV2 infection is considered ubiquitous, and since most pigs are vaccinated against PCV2 at an early age, majority of the farmed pigs in developed countries have antibodies against PCV2.

Neutralization assay to detect PCV2-specific antibodies is a technique of high utility, but it is prolonged and laborious and requires fluorescent labelled antibodies and a cell culture capable laboratory to perform the assay. For the routine and rapid diagnosis of samples, conventional polymerase chain reaction (PCR) assays followed by RFLP is helpful. The discovery of PCV2 as a pathogen, and that of PCV2b as an emergent genotype, were triggered by variations in RFLP patterns [1,72]. The latter was a change from RFLP type 422 to RFLP type 321. PCR followed by Sanger sequencing has greatly contributed to the accumulation of the current wealth of knowledge on the molecular epidemiology of PCV2. However, similar to seroconversion to PCV2, the ubiquitous and enzootic nature of PCV2 makes this assay redundant in the current setting. The PCR assay has been superseded by quantitative PCR (qPCR) assays to estimate the viral genome copy numbers as a measure of the viral load in body fluids and tissue samples. The qPCR is of high utility in detecting the etiology during laboratory disease investigation, especially in cases of reproductive failure where the viral load in fetuses is a very useful indicator. Another variation of this technique is based on fluorescent probe-based PCRs, such as Taqman probes, which enable to specifically detect PCV2 genotypes and also allow for the detection of multiple genotypes of PCV2 and other pathogens in a single reaction. Automated sample processing and fluorescent probe-based PCR assays have revolutionized the diagnostic capacity and information available to investigate clinical cases. Unlike PCR-based assays, which need prior sequence information and primers, next generation sequencing techniques (NGS) have made it possible to obtain information on PCV2 molecular epidemiology along with co-infecting pathogens and also have enabled the unmasking of any potential associations not hitherto known. The PCV2 research community is yet to catch up with this technology, reflected by the scarcity of reports in the public domain utilizing this technique [87].

In light of the current scenario, where the genotype prevalence switch from PCV2b to PCV2d is thought to be occurring worldwide, it is important to develop assays capable of detecting and measuring changes in the immune response. ELISA-based assays to differentially identify the genotype of infecting PCV2 would be of high utility. Current methods of utilizing sequence information to study the molecular epidemiology do not enable the direct understanding of changes in immunological parameters of the emerging genotype(s). Developing serological assays to study changes in the neutralization profiles in the serum of PCV2 infected or vaccinated pigs, such as immune response against the decoy epitope [82], is essential. Utilization of PEPSCAN, phage display or protein array-based serological assays at a sufficient resolution to quantify immune response against specific epitopes of the PCV2 capsid protein could improve our understanding of the interplay between different PCV2 vaccines and genotypes [74,95,96,108]. Development of competitive blocking ELISA with panels of specific peptide epitopes from the capsid protein of different PCV2 genotypes and complimentary monoclonal antibodies would enable precise monitoring of the shifts in epitope-specific immune responses. Accumulation of more knowledge on neutralization profiles of the different PCV2 genotype capsids will help this effort. Establishment of a dedicated, peer-reviewed, curated database for PCV2, similar to the PRRSV and influenza virus databases, is essential to bolster the efforts of the PCV2 research community.

## 6. Conclusions

PCV2 made a dramatic appearance towards the end of last century and soon became recognized as the most important pig pathogen, which was followed by the rapid development of successful vaccines, utilizing multiple approaches ranging from inactivated PCV2 vaccines to baculovirus expressed recombinant capsid protein-based vaccines. Until a few years ago, PCV2 was considered a successfully controlled emergent pathogen. However, the worldwide second genotype shift to PCV2d and a debated increase in associated virulence and vaccine failures has raised alarm. As a biological problem, PCV2 is a fascinating puzzle of minimal dimensions, which is not yet completely unraveled. Unambiguously deciphering its molecular evolution and epidemiology is a daunting task, given its ubiquitous nature and expanding genetic spectrum. Understanding the forces behind the emergence of PCV2d as the most prevalent genotype is of prime importance. The recent report of porcine circovirus 3 (PCV3) in the US [124,125], with a 2000 bp genome, is probably an indication of the plasticity of the circoviral genomes. The use of vaccines in PCV2 is perhaps one of biggest success stories in veterinary vaccines; however, it is clear that vaccines are no replacement for good biosecurity programs in intensive pig husbandry.

## Figures and Tables

**Table 1:** Estimated time of origin of the main porcine circovirus type 2 (PCV2) genotypes and earliest specimen retrieved.

Genotype	Estimated Time of Divergence (Reference)	Earliest Archived Tissue Identification
PCV2a	1966 (1945–1983) [60]	1962 [68]
	1964 (1948–1974) [55]	
PCV2b	1989 (1980–1995) [60]	1979 [69]
	1973 (1952–1996) [55]	
PCV2d	1986 (1971–1996) [58]	Genbank Accession Number JX512856, 1999 from a healthy herd [52,58]

**Table 2:** Comparison of viral load inhibition by heterologous PCV2 genotype vaccines.

Study No.	Vaccine Genotype	Challenge Genotype	Co-Infecting Agent, If Any	Comments on Viremia
1	PCV2b	PCV2a	None	Viral load in lymph nodes of vaccinated pigs at 21 days after challenge with PCV2a was higher compared to pigs challenged with PCV2b (statistical significance not known) [109]
		PCV2b	None	
2	PCV2a	PCV2b	PRRSV, PPV	Compared to unvaccinated control pigs, the inhibition of serum viral load after challenge was 25% with the PCV2a vaccine and 100% with the PCV2b vaccine [78]
	PCV2b			
3	PCV2a	PCV2d	PRRSV	92.2% inhibition of serum viral load with a PCV2a vaccine and 100% inhibition of serum viral load with a PCV2d vaccine on day 49 after challenge [113]
	PCV2d			
4	PCV2a	PCV2b	None	Vaccinated challenged and vaccinated contact pigs displayed approximately one log decrease in viral load; however, the viral load was higher than 10^4^ genome copies per mL of serum until 42 days after inoculation [115]
5	PCV2b	PCV2b	None	One out of the five pigs vaccinated with a PCV2d vaccine displayed detectable viral load at 21 days after challenge, compared to none in the PCV2b vaccine group [118]
	PCV2d			
6	PCV2a	PCV2d	None	Viral load after PCV2d challenge was reduced by one log or more in vaccinated pigs; however, serum viremia and shedding of virus were observed at 21 days after challenge in vaccinated pigs [110]

PRRSV: Porcine reproductive and respiratory syndrome virus; PPV: Porcine parvovirus.
